# Progress in metathesis chemistry

**DOI:** 10.3762/bjoc.6.124

**Published:** 2010-11-23

**Authors:** Karol Grela

**Affiliations:** 1 Faculty of Chemistry, University of Warsaw, Żwirki i Wigury Street 101, 02-089 Warsaw, Poland; 2 Institute of Organic Chemistry, Polish Academy of Sciences, Kasprzaka 44/52, 01-224 Warsaw, Poland

In contrast to the production of simple bulk chemicals and polymers, which started shortly after the discovery of the “olefin scrambling” reaction, it was not until much later that this transformation was “rediscovered” by the academic world. In the last decade of the 20^th^ century, the so-called olefin metathesis reaction gained real significance in advanced organic synthesis [1]. The development of well-defined catalysts and an understanding of the reaction mechanism prompted an extraordinary scientific turnaround, revolutionizing retro-synthetic planning in total synthesis groups and in medicinal chemistry R&D laboratories around the world. The importance of “the development of the metathesis method in organic synthesis” has been fully recognized by the award of the Nobel Prize in Chemistry for 2005 jointly to Yves Chauvin, Robert H. Grubbs, and Richard R. Schrock [2].

Organic chemists are now provided with molybdenum and ruthenium complexes that present good application profiles for a range of metathesis reactions. Most of the ruthenium initiators can be handled in air and are compatible with functionalized substrates, such as unsaturated esters, amides, ketones, aldehydes and even alkenes bearing protic functions, e.g., hydroxy or carboxylic acid groups. While more sensitive towards air and moisture, molybdenum catalysts provide excellent results in stereoselective metathesis and are sometimes more active. Therefore these catalyst families can be used in a complementary fashion. Currently, olefin metathesis (and its sister reaction, alkyne metathesis) [3] is one of the most intensively studied transformations in synthetic organic chemistry, as witnessed by a veritable “explosion” in the number of published syntheses where metathesis is the key step. Despite its power and applicability, metathesis is still experiencing some growing pains outside academic laboratories and the industrial production of simple chemicals. It is surprising that while this transformation has been used in laboratory scale syntheses of thousands of natural and biologically active compounds, it has not yet entered commercial pharmaceutical manufacturing. This could be due to a variety of reasons, such as the still not perfect stability and sometimes low activity of existing catalysts, problems with removing/recycling of catalysts after the reaction, unfavorable patent situation and licensing strategies etc. [4]. Fortunately, the international community of chemists, fully aware of the above limitations, is working hard to develop new metathesis catalysts and strategies in order to solve some of the existing problems. The enormous progress which has been made over last few years provides an equally impressive lesson about the chemist’s ability to innovate. The variety of metathesis catalysts now available on the market as well as the number of commercial players involved, are increasing. New applications of this methodology are being developed, including sustainable production of valuable chemicals from renewable sources. The importance of these developments is now widely appreciated and more and more commercial applications will surely follow.

The papers collected in this Thematic Series nicely highlight many of the aspects mentioned above and will certainly help to update the “state of the art” knowledge in this fascinating field. In this Thematic Series contributions from many of the leading practitioners in the area, which cover a wide range of topics related to alkene and alkyne metathesis, are presented. It is therefore a pleasure to serve as Guest Editor for this Thematic Series in the *Beilstein Journal of Organic Chemistry*, on “Progress in metathesis chemistry”. I would like to thank all authors for their excellent contributions. Enjoy reading them!

Karol Grela

Warsaw, November 2010
